# The current situation of human resources for health in the province of Cabinda in Angola: is it a limitation to provide universal access to healthcare?

**DOI:** 10.1186/s12960-017-0255-7

**Published:** 2017-12-28

**Authors:** Damas Macaia, Luís Velez Lapão

**Affiliations:** Global Health and Tropical Medicine, Instituto de Higiene e Medicina Tropical, Universidade Nova de Lisboa, Lisbon, Portugal

**Keywords:** Human resources for health, Planning, Policy-making, Universal access to healthcare, Cabinda/Angola

## Abstract

**Background:**

Angola is among sub-Saharan African countries dealing with a crisis of Human Resources for Health (HRH). The province of Cabinda, besides the efforts, still suffers from both HRH shortage and a badly distributed health workforce. In Cabinda, one can find urban concentration and rural shortages of healthcare professionals, many rural areas’ healthcare facilities often secured only by basic or medium level HRH; and difficulties in developing HRH retention strategies in rural areas where most services are covered by foreign HRH. This study aims at analysing the situation of HRH in the province of Cabinda. It considers organizational issues, policies and practices resulting from the HRH strategy followed in the recent years, moreover the creation of a medical school. The context that affects the distribution of the health workforce is analysed to contribute to the development of evidence-based policies that promote a better HRH allocation in the poorest and distant villages in the province.

**Methods:**

A mixed-methods study was developed, combining a quantitative and qualitative approach to analyse HRH situation in the province of Cabinda. Data was collected from key informants, selected by intentional sampling from public and private health organizations, to respond to a questionnaire and a semi-structured interview. Quantitative and qualitative data was analysed with descriptive and inferential statistics and content analysis respectively. The study was complemented by a comprehensive desk review.

**Results:**

Results show a clear change in HRH data from 2011 to 2015 with significant fluctuations due to variations in retirement, migration and lack of regular public HRH recruitment tenders. HRH density is apparently better in rural when compared with urban areas. However, one should bear in mind that often HRH allocated to rural areas do not stay there, which leads to real geographical imbalances. Factors like lack of proper incentives for HRH retention and social support goes against significant HRH management efforts contributing to this result. Whereas HRH are financed by the State General Budget, the majority of health facilities are still dependent on the Provincial Health Secretariat budget.

**Conclusion:**

The study provides a broader view of the current HRH situation in Cabinda Province. Geographical imbalances and other issues with impact in delivering universal access to healthcare are highlighted.

## Background

Health service productivity depends mainly on the size, skills and commitment of its workforce [1]. Human Resources for Health (HRH) are widely recognized as the most important assets and the pillar of any health system for the provision, production and delivery of healthcare services [2], thus being one of the focus areas for the strengthening of health policies [3].

Many countries, especially middle- and low-income ones, are facing serious shortages of HRH, being this a major obstacle to achieve their health objectives [1, 4]. The World Health Organization (WHO) in the 2006 report “Working together for health” identified a global shortage of almost 4.3 million physicians, midwives, nurses and support workers. It also identified the 57 countries with critical shortages of these HRH, 36 of these located in Africa [3].

Angola is among the countries with critical shortage of HRH [4–8]. Cabinda, the subject of this study, is one province of Angola with acknowledged shortage and maldistribution of health workforce. It seems to include a larger urban concentration and significant rural deficits of HRH. Most rural health facilities are secured only by a limited number of HRH on primary and secondary care level facilities and face huge difficulties in attracting and retaining HRH in these areas [9, 10].

The Medical School of the University of Cabinda was established in 2007. In 2013, Cabinda healthcare services received the first group of 48 locally graduated physicians [11]. In 2015, arrived the first 43 nurses [12] trained in the local public University. Every year an additional number of nurses, although a tiny number, graduated from the private Polytechnic Institute of Cabinda. The training of diagnostic and therapeutic technicians is also provided by both the local public University and by the private Polytechnic Institute.

The actual distribution of these resources is a key challenge for the urban-rural balance of available HRH. In 2014, the province had only 47 national physicians (Angolan) [13] working in public healthcare services. Urban areas, covering 86.9% of the population [14], held approximately 79% of these physicians, with the remaining 21% distributed in the rural municipalities, yet mostly of the latter occupying management functions in healthcare units. The same situation also occurs at the national level: in 2011, about 42% of physicians were concentrated in the country’s capital (Luanda), which had only an estimated 24% of Angola total population. The total of provincial capitals concentrate about 85% of physicians’ workforce [15].

The debate on the distribution of HRH falls within the scope of planning and management of HRH, including aspects such as the mobilization of personnel through strategies for attraction and retention in rural and remote areas [16], for instance, the example of Zambia, which in 2003 developed a positive experience of providing additional financial incentives to those that accepted working in those remote places [17].

For the development of HRH attraction and retention strategies, WHO has proposed a set of global recommendations that include four categories of coordinated interventions: education, regulation, financial incentives, and professional and personal support [18]. Thus, identifying and developing interventions to support the geographical balance of the health workforce requires a comprehensive understanding of HRH current situation and of the current management and planning practices [18–20], as well as the factors that influence the HRH decisions to accept, or not, working in a remote area, especially in the context of medium- and low-income countries [18]. Furthermore, regarding human resources management, it is also important to understand management and performance improvement; job market; education, training and research; priority health programs; and monitoring and evaluation issues [21].

The present study aims at analysing the current situation of HRH at the province of Cabinda. It considers organizational issues, policies and practices resulting from HRH strategy. The context that affects the distribution of the health workforce and the HRH management practices is assessed to contribute to the discussion, and the construction, of recommendations to improve the geographical distribution of HRH in Cabinda.

## Methods

This research uses mixed methods, combining quantitative and qualitative approaches to analyse HRH in Cabinda, including policy, strategy, planning, finance, education and management systems aspects [4, 21, 22]. Data was collected from a questionnaire and a semi-structured interview. For the analysis of quantitative data both descriptive and inferential statistics was used, supported by software tools (SPSS version 23, and Excel 2013). For the qualitative data, content analysis was used [22] to better understand the many dimensions of the problem [23]. This information was complemented by a comprehensive desk review: It includes seven Health Provincial Secretary reports, two University reports, four National Decree laws, two Working reports and one National Plan from the Angola’s Ministry of Health. The data collection occurred from February to March 2015.

The questionnaire was adapted from WHO and it provides a quick assessment of HRH in countries dealing with HRH shortages [21].

The questionnaire includes open and closed questions. The quantitative data was analyzed statistically, testing the equality of means, covering both key informants from rural and urban areas. The *H*_0_, the null hypothesis, where *H*_0_: *μ*1 = *μ*2, suggests that both urban and rural healthcare personnel were equally distributed. The *H*_1_ is the alternative hypothesis, where *H*_1_: *μ*_1_ ≠ *μ*_2_,*μ*_1_—urban area healthcare personnel number mean; *μ*_2_—rural area healthcare personnel number mean), and *t* test rule, reject *H*_0_ if *p* value < *α* = 0.05. The questionnaire was applied to key informants selected by intentional sampling, from public and private health organizations. The sample included 61 organizations (about 28% of the total) and 25 semi-structured interviews addressed to key informants. The interview sample size was defined by the theoretical saturation criterion [24].

The identification of study participants were codified: INF1, INF2 ... INF61 was used for the questionnaire participants and INT1, INT2... INT25 was used for interviewed participants.

The protocol of this study was approved by both the Ethics Committee of the Institute of Hygiene and Tropical Medicine, Nova University of Lisbon, and the health authorities in the province of Cabinda.

## Results

The study included key informants from 57 health organizations (HO) and 4 teaching organizations. 60.7% of these HO are located in urban area and 39.3% in rural area; 70.5% are public, 26.2% private and 3.3% military.

These participants had, on average, 46 years old; 68.9% were male and 31.1% female; 13.1% were physicians, 63.9% nurses, 6.6% diagnostic and therapeutic technicians (DTT) and 16.4% from other professions. From these 61 key informants, 25 were interviewed afterwards, 20% of which work on HRH planning, 4% were top decision-makers, 16% were HRH trainers and 60% were HRH.

### Cabinda health service characterization

**Fig. 1:**
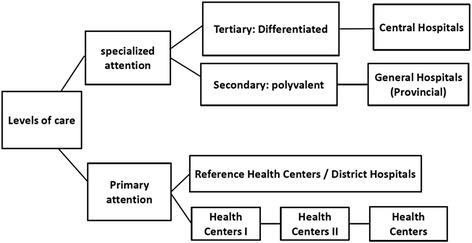
Health Service Delivery Levels in Angola. Source: Ministério da Saúde, 2009 [27]

The desk review provided most of the information to characterize Cabinda’s health system, whereas interviews enabled to validate it and to obtain complementary data. The Cabinda province health system is structured according to Angolan National Health System (NHS) [25]. The NHS is structured in three levels of care (Fig. 1): The first level, comprising healthcare centers, reference healthcare centers, district hospitals, nursing stations and physicians’ offices provides low complexity services; the second level includes general hospitals, which provide intermediate-complexity services; and the third level comprising central hospitals and specialties [26].

Regarding management, the healthcare services provided to the population in the province depends on the Provincial Health Department, which is a governing body with administrative subordination to the Governor of the Province, and technical subordination to the Minister of Health. In the municipalities, healthcare services are managed by the health department, with technical subordination to the provincial secretary of health and administratively to the municipal administrator [27].

### Public health network

In the province of Cabinda, public health network integrates Health Units (HU) from both the first and second levels, distributed as follows (Tables 1 and 2):

**Table 1:** Distribution of public health units in the province of Cabinda in 2014

Municipalities	Hospital units	Healthcare centers	Health posts	Subtotal
Cabinda (provincial capital)	4	13	29	46
Cacongo	1	2	17	20
Buco-Zau	2	2	19	23
Belize	1	3	9	13
Total	8	20	74	102

Source: Cabinda’s Provincial Health Secretary Report 2014 [13]

**Table 2:** Distribution of private health units in the province of Cabinda in 2014

Unit type	Number
Clinics	8
Medical centers	44
Doctors’ office	1
Medical posts	61
Clinical analysis laboratories	4
Total	118

Source: Annual Report of the Inspection Department, Cabinda’s Provincial Health Secretary, 2014 [34]

The province’s public health network includes 102 healthcare facilities of various categories (hospitals, health centers and health posts). The capital of the province concentrates four hospitals, while the interior municipalities have one municipal hospital each. The municipality of Buco-Zau also houses a regional hospital, for specialized care, covering also the north municipalities of Belize and Buco-Zau. Other smaller units are distributed in all municipalities, again with the highest concentration in the capital.

The province’s private healthcare services are characterized by 118 different types of health facilities, from clinics, medical centers, physicians’ offices to clinical laboratories.

### Evolution of HRH from 2011 to 2015

The evolution of HRH in the public sector, from 2011 to 2015 (Table 3), presents significant fluctuations, with significant ups and downs in the number of physicians and DTT, a constant decline in the number of nurses and a considerable increase in administrative and hospital support staff. However, the overall number of healthcare professionals (physicians, nurses and DTTs) have actually decreased about 7% from 2011 (1854) to 2015 (1725).

**Table 3:** Evolution of HRH in the public sector in Cabinda in the last five years (2011–2015)

year	Physicians			Nurses	Diagnostic and Therapeutic Technicians (DTT)	Administrative staff and hospital support	Total
	National	Foreign	Subtotal				
2011	48	84	132	1 347	375	732	2 586
2012	45	104	149	1 341	366	600	2 456
2013	51	86	137	1 267	367	905	2 676
2014	47	95	142	1 261	355	937	2 695
2015	52	99	151	1 235	373	861	2 620

Source: Adapted from the Cabinda’s Provincial Health Secretary Reports 2011 [35], 2012 [36], 2013 [29], 2014 [13], and 2015 [37]

The fluctuations vary about 8% (in national physicians) and 24% (in foreign physicians). This phenomenon can be mainly attributed to HRH issues (e.g. recruitment and retirement factors) that, in a context of HRH shortage, are very relevant while addressing healthcare service organization and the response to the population demands.

### Distribution of HRH

Table 4 shows the distribution of healthcare workers in the public sector at the municipality level in 2014. The province’s capital Cabinda holds 79.5% of workers. The preliminary data from 2014 Census shows that Cabinda is the most densely inhabited city with 86.9% of population [14]. The major shortages were identified on the other municipalities that have only 21.5% of health workers, where a significant number of these HRH occupy management positions (i.e. not providing healthcare services).

**Table 4:** Distribution of HRH in the province of Cabinda by municipalities in 2014

Municipalities	Physicians			Nurses	DTT	Hospital administrative and support staff	Subtotal	
	National	Foreign	Subtotal				Number	Percentage
Cabinda	38	69	107	981	301	752	2 144	79.5
Cacongo	4	5	9	115	21	50	195	7.2
Buco-Zau	3	19	22	89	21	92	225	8.3
Belize	2	2	4	76	12	43	135	5
Total	47	95	142	1 261	355	937	2 695	100

Source: Cabinda’s Provincial Health Secretary Report 2014 [13]

### Density of HRH

The health professionals’ densities are presented in Table 5, showing particularly low physicians and DTT ratios per 10,000 inhabitants. Still, compared to the national average, Cabinda’s province gets higher ratios than national values in all categories of health professionals. In total, combining together physicians, nurses and DTTs, Cabinda’s ratio of health professionals per 10,000 inhabitants reaches 25 (Table 5). This value, although above Angola’s average, is still half way from the WHO targets of 44.5 skilled health professionals per 10,000 inhabitants [18]. Apparently higher densities of nurses are observed in the rural municipalities. The problem is that part of these nurses are often not actually working in these rural areas. The interviews showed that some nurses working in the most remote places, often associated themselves into small teams, have “legitimized” an unconventional working shift. The unconventional working shift is organized in the following way: For each team, one nurse will turn on the rural site for a week while the others will be working in the city. On the next week, the shift changes and another member of the team will be then working in the remote place. With this subterfuge, the healthcare units in remote places cannot fully rely on all their staff, which means that the actual density is much lower than the official one.

... We do not live here, all colleagues live in the city ... and here we are just staying for one week ... (ENT7)

**Table 5:** HRH density at the provincial level in 2014 and National in 2013

Municipalities	Population	Physicians		Nurses		DTT	
		Number	Ratio p/10 000 inhabitants	Number	Ratio p/10 000 inhabitants	Number	Ratio p/10 000 inhabitants
Cabinda	598 210*	107	1.7	981	16	301	5
Cacongo	36 778*	9	2.4	115	31	21	6
Buco-Zau	33 843*	22	6.5	89	26	21	6
Belize	19 454*	4	2.0	76	39	12	6
Total provincial	688 285*	142	2.0	1 261	18	355	5
National level (2013)**	20 734 410	2 045	1.0	32 177	16	6 255	3

Source: Adapted from the Secretaria Provincial Health Report (2014) [13]

*INE (2014) [14]

**Costa & Freitas (2014) [15]

### Policies, regulation and planning

Throughout the interviews, it was found that Cabinda’s province health director has, since 2014, been developing a HRH strategic planning. This HRH strategic planning includes the preparation of health facilities’ plans to define the quantity and quality of health unit services required and the associated HRH to provide those services, in order to be considered in the General State Budget (GSB).

... in terms of strategies for the development of HR ... in 2014 the proposals that we have worked out ... appear in this strategy ... We are now in a process of drawing up lists of personnel (HRH) requirements.... (ENT17)

In 73.8% of the units surveyed, informants said they still do not have concrete HRH plans; 59% reported no organizational regulations; and 32.8% mentioned having health professionals not properly registered within the respective professional bodies. This shows that HRH planning and regulation are not taken seriously in the public health system. A slightly better situation can be found in the private sector where 60% of the units’ informants reported having regulations and 68.8% reported having enrolled personnel in their respective professional bodies.

### HRH financing

Public health services are financed through the Angolan state budget. Additionally, there are some significant support from the provincial government budget, non-governmental organizations (NGOs), and some companies that support some vertical health programs. However, 62.3% of the units’ surveyed informants reported not having received their own part of the state budget, with 49.2% of those directly dependent on the Provincial Department of Health. This means even more difficulties in staff recruitment and retention, and incapacity of most of these units to develop their own initiatives, to motivate and retain health professionals. The planned decentralization of health services is expected to allow each municipal Health Departments to use the financial resources of the Municipal Administrations, which eventually will strengthen the capacity of these municipalities to improve the retention of their employees through their own initiatives with non-financial incentives to motivate workers.

### Management and performance improvement

The imbalance and poor distribution of HRH between urban and rural zones was recognized by participants: In 83.3% of the healthcare units located in the rural areas, informants reported not having enough staff to meet the needs of healthcare, while 51% of informants in urban areas believed to have enough staff to meet their needs.

However, the statistical tests for equal means for HRH coverage comparing data from urban and rural areas showed significance < *α* = 0.05 for all categories of staff: physicians (0.016), nurses (0.022), DTT (0.048) and hospital administrative and support staff (0.022), which rejects the *H*_0_. Hence, there is no statistical evidence showing that the two means are not identical, suggesting that the HRH policy approach for the two zones has indeed identical coverage.

Regarding the recruitment of new workers, in 70.5% of the reporting units, it was done through public tender or transferring. The process is managed by the Human Resources Services at the Provincial Health Department, which after the public tender is complete, assigns the new works to their locations. However, 26.2% of the units said that they hire new staff through local recruitment (see that this procedure also takes place in private units); 3.3% used other forms of recruitment (a procedure often used by the military medical units).

The physician’s average working hours in the urban units is 39.7 h per week (~ 8 h/day); the nurses about 39.7 h (~ 8 h/day); the DTT about 40.1 h (~ 8 h/day) and hospital administrative and support staff approximately 37.1 h (~ 7 h/day). On the other hand, the physicians’ working hours in rural zones are on an average 21.7 h (~ 4.5 h/day), nurses 57.2 h (~ 11.5 h/day), DTT 26.5 h (~ 5.5 h/day) and the administrative and hospital supporting staff about 29.4 h (~ 6 h/day).

As far as the health units surveyed, 32.8% of the informants reported having staff on a part time basis and the vast majority of private units (62.5%) work with staff in the same situation. In fact, 54.1% of all health units have a staff in dual employment status. The public sector has 46.7%, and the private ones have 75% of its health units working with personnel in dual employment. However, 79% of professionals, who are also active in other institutions, have no authorization to do so.

The level of employee satisfaction was also addressed. 45.9% of the units received a good evaluation, 39.3% a moderate, 13.1% a lower and only 1.6% received a higher evaluation. In urban zones, the staff satisfaction level was considered good in 33.3% of the units, 45.8% moderate and low on 20.8%. It was found that 62.2% of the units do not have non-financial incentives to motivate their staff, and in the rural zones, it reaches 70.8% of the units. The reasons for staff dissatisfaction are mainly related with the social and working conditions, low wages, lack of incentives, excessive time spent in rural areas and the fact that organizations are understaffed.

The level of the users’ satisfaction was good in 68.9% of the units, moderate in 19.7%, higher in 6.6% and lower in 4.9%.

Regarding monitoring workers’ performance indicators, the vast majority of units (95.1%) do not have it and 4.9% (e.g. in some private units) consider productivity and interpersonal relationships as a criteria to monitor the performance of their staff.

### Health professionals’ training

Within the process of expansion of the higher education network in the country, the province of Cabinda, together with Zaire (the nearby province), benefit with a new public university that currently offers, among other graduations, Medical School, Nursing degree, Clinical Analysis and Clinical Psychology. The private higher education sector also offers a degree in Nursing, and the health technical training schools (one public and another private) are forming medium-level health technicians from different specialties. The access to these degrees is based on exams and all applicants are treated equally, without distinction for their origins. To determine the number of places for admission to educational institutions, it is worked with partners so that the needs of the province are taken into account.

... At the provincial level we work with many partners ... for the definition of needs ... during the application period, there cannot be partial, they treat everyone the same way … (ENT18)

The educational curricula were determined centrally and used nationwide. However, with the expansion of higher education in the country, a new protocol was signed with Cuba, through which medical schools, nursing and others are operated by Cuban professors and the curricula included in the protocol are taught.

In the first year of the medical school, 78 students were registered [28], and 48 finished successfully (about 62%) in 2013 [11]. A similar number have been observed in the following years.

## Discussion

Data collected about the current situation of HRH is important for identifying the contextual factors affecting the distribution of these resources. These factors provide the basis for the future development of interventions aimed at improving the geographical balance of the workforce in the province of Cabinda. Overall, a slight decrease in HRH in Cabinda was identified, from 2011 to 2015. As a consequence of the shortage of Angolan health professionals, the country has been relying on the international cooperation agreements with other countries (e.g. Cuba, North Korea, Russia, Vietnam) to cover its needs, as seen in Table 4. The results show an important variation in foreign physicians. Therefore, these cooperation agreements bring benefits to health services; however, it has also negative implications especially in communication with patients due to language and culture barriers. At the same time, after ending the contracts of these professionals, they do not always have immediate replacements or are covered by others national HRH. As the country is nowadays facing financial crisis (i.e. corresponding to a lower number and frequency of public tenders), this variation of foreigner HRH can even have a greater impact with clear consequences, particularly in the access to health professional and quality care.

One significant result was the fluctuation observed over the years on the number of HRH available, with ups and downs in the numbers of physicians and DTT (Table 3), that seem to be associated more with the physicians’ recruitment patterns and international cooperation agreements with other countries. This can be also perceived at the Provincial Health Secretary report [29]:

There was a decrease of ... physicians overall. This can be justified by the departure of physicians ... resulting from the termination of service agreements with the Provincial Government.

The impact of HRH graduated from a local university could be very positive but is only starting. The first 48 graduated physicians from the local Faculty of Medicine since it opened in 2013 and most of the other graduates from 2014 and 2015 are not yet fully working in the public service carrying out their activities as physicians. Most of them are still waiting for the opportunity to respond to a public tender. This also reflects the changes observed in the development of the number of physicians (Table 2) and the low density of these professionals (Table 5).

The circumstance of low physician per 10,000 inhabitants ratios, although apparently better in the rural municipalities, compared to the capital city, is due to the fact that most of these physicians in these municipalities are playing the role of manager of health services or health facilities, i.e. are not seeing patients. The constant declines observed in the evolution of the nurses (Table 2) could be caused by staff migration to other sectors or to other provinces.

It was also perceived that the densities of nurses are apparently better in the rural areas (Table 5) where these nurses are somewhat self-organized into teams (rounds) working on a rotating basis. This is a strategy they use to solve the constraints caused by the use of unattractive areas and staying away from their families. This strategy to “legitimize” a non-legal behaviour by the decision maker can be interpreted in the light of the Rational Choice Theory, featuring individuals as rational subjects, choosing actions able to maximize their own individual interests. A rational being is someone who has goals and beliefs he/she searches and chooses actions in the light of these dimensions [30]. Thus, individuals act rationally by trying to achieve a more beneficial balance between costs and rewards, involving some kind of social exchange (goods and services) or social interaction that includes exchanging approval and other behaviours valued [31].

The weekly working hour pattern suggests that people in rural and remote areas have less access to physicians, DTT and administrative workers and hospital support, whose average hours of service are very low, opposite to nurses with larger working hours, showing that the units located in these areas are better provided by nurses but where the doctor’s presence is still not a reality [9, 10]. The nurse is often the only worker in these health centers [6]. This contrasts with the health units in the urban area where the average working hours for all professional categories are within the established by law (8 h a day and 40 h a week [32]). This issue also contributes to the existing geographical imbalance of health workforce distribution in Cabinda’s province.

The dual employment in the province is significant and most health units of the private sector (62.5%) work with professionals who have a permanent employment in the public service. This phenomenon is well-known in Africa [33]. Low income was identified as one of the personal reasons for dissatisfaction, especially in the rural areas, taking into account that wages differ only by workers’ careers and categories, regardless of the geographical location of employment.

Public service recruitment is done centrally. Health professionals often refuse working in rural areas when they are placed or transferred there. They usually seek alternatives to circumvent the situation. Consequently, many professionals remain in rural areas for longer periods without replacement.

... in the recruitment interview I was told to come here for two years ... I am already nine years over the set time. (ENT4)

As far as it is known, the province of Cabinda has no clear strategy for attracting and retaining HRH needed in the rural areas. Several neighbour countries and regions are experiencing different strategies to attract and retain HRH for the most deprived areas, like the example of Zambia, which in 2003, has developed an experience of providing additional financial incentives and other facilities to HRH to those that accept working in those places. This and other examples should be considered in the creation of policies focused on universal health coverage.

The inferential study shows a balance in the HRH policies. This balance can be understood as a result of the centralized recruitment process (via Provincial Department of HR) for public services that allocates HRH to all health units, as well as from the salaries that are not differentiated in terms of geographic location. However, although there is no statistical difference between rural and urban areas, the interviews show a different reality, clearly identifying a significant imbalance in rural areas. This reality needs to be better understood and policies need to be drawn to tackle it.

## Conclusion

From this study, it is clear that, although experiencing a situation above the average in Angola, resulting from significant HRH management efforts, Cabinda is still a province living with shortage and poor distribution of the workforce. In spite of the Cabinda health administration following a balanced HRH policy (i.e. the null hypothesis was correct), both surveys and interviews have identified some geographical imbalances between rural and urban areas, and other HRH issues with direct impact on the healthcare access in Cabinda. This shows that although HRH is recognized as one important pillar of the Angolan health system, the HRH weaknesses in the province make it hard to positively contribute to the universal health access.

This study allowed a broader view on the current situation of HRH in the province, with the results showing contextual factors underlying imbalances such as real lower density of health care professionals, an evolution, over the years, of HRH with fluctuations in numbers due to factors like migration, financing bottlenecks and foreigner HRH contracts’ expiring. The difficulties in managing HRH is a central problem, leading to lack of regular tendering; fragile planning and regulation practices; lack of incentives and conditions for attraction and retention of health professionals in the most deprived areas and lack of proper mechanisms for obtaining quality information for HRH monitoring. To solve these problems, policy-makers need to address them seriously, primarily by selecting policies that, based on this research and other information, would contribute for improving working conditions and leveraging the new supply of physicians in deprived areas.

## Abbreviations

DTT: Diagnostic and therapeutic technicians
HRH: Human Resources for Health
HU: Health Units
INF: Informant
INT: Interviews
NHS: National Health System
SGB: State General Budget
SPSS: Statistical Package for Social Sciences
WHO: World Health Organization
